# The Dorsal Mesenchymal Protrusion and the Pathogenesis of Atrioventricular Septal Defects

**DOI:** 10.3390/jcdd3040029

**Published:** 2016-09-26

**Authors:** Tara Burns, Yanping Yang, Emilye Hiriart, Andy Wessels

**Affiliations:** 1 Department of Regenerative Medicine and Cell Biology, Medical University of South Carolina, 173 Ashley Avenue, Charleston, SC 29425, USA; burnsta@musc.edu (T.B.); yyp524@163.com (Y.Y.); hiriart@musc.edu (E.H.); 2 Department of Histology and Embryology, Shanxi Medical University, No 56 Xin Jian Nan Road, Taiyuan 030001, Shanxi, China

**Keywords:** atrioventricular septal defects, AVSD, dorsal mesenchymal protrusion, DMP, pathogenesis, etiology, congenital heart defect, development

## Abstract

Congenital heart malformations are the most common type of defects found at birth. About 1% of infants are born with one or more heart defect on a yearly basis. Congenital Heart Disease (CHD) causes more deaths in the first year of life than any other congenital abnormality, and each year, nearly twice as many children die in the United States from CHD as from all forms of childhood cancers combined. Atrioventricular septal defects (AVSD) are congenital heart malformations affecting approximately 1 in 2000 live births. Babies born with an AVSD often require surgical intervention shortly after birth. However, even after successful surgery, these individuals typically have to deal with lifelong complications with the most common being a leaky mitral valve. In recent years the understanding of the molecular etiology and morphological mechanisms associated with the pathogenesis of AVSDs has significantly changed. Specifically, these studies have linked abnormal development of the Dorsal Mesenchymal Protrusion (DMP), a Second Heart Field-derived structure, to the development of this congenital defect. In this review we will be discuss some of the latest insights into the role of the DMP in the normal formation of the atrioventricular septal complex and in the pathogenesis of AVSDs.

## 1. Introduction

### 1.1. Atrioventricular Septal Defects in Humans

Atrioventricular septal defects (AVSDs) form a class of congenital heart malformations found in 1 in 2000 live births [1] and in approximately 5% of all persons suffering from congenital heart disease (CHD). AVSDs are particularly prevalent in the spectrum of defects associated with genetic disorders such as Heterotaxy Syndrome, where they are found in approximately two-third of all cases, Down Syndrome (DS), where they are observed in 20%–25% of patients, Smith-Lemli-Opitz syndrome (SLOS) were they occur in 20% of all patients, and patients with Ellis-van Creveld and Noonan syndrome [2,3]. When AVSDs are found in isolation they are referred to as nonsyndromic AVSDs (NSAVSDs). Over the years, numerous genes have been linked to syndromic and nonsyndromic AVSDs. These include ALK2 [4], ALK3 [5], BMP4 [6], CEP152 [5], CHD7 [5], CRELD1 [7,8], GATA4 [9], MDM4 [5], NIPBL [5], NKX2.5 [10,11], TBX5 [12], and ZFPM2 [5]. It is important to point out, however, that in many cases it is “guilt by association” and that the molecular and cellular mechanisms linking the gene mutations with the pathogenesis of the AVSDs remain to be elucidated. In addition, non-genetic risk factors have also been linked to AVSDs. In a large case-control study, conducted to assess risk factors for congenital cardiovascular defects (the Baltimore–Washington Infant Study) invaluable information was provided regarding the contribution of non-inherited risk factors to the etiology of AVSDs. Thus, the study showed that pre-gestational diabetes significantly increases the risk of both syndromic and non-syndromic complete AVSD [13,14], that urinary tract infection during the first trimester of pregnancy is a risk factor for AVSD [15], and that maternal use of ibuprofen, moderate to heavy maternal cigarette use, maternal use of cocaine, and maternal exposure to paint, varnishes, and ionizing radiation are implicated in the etiology of the defect [16,17].

### 1.2. AVSDs—Subtypes

**Figure 1:**
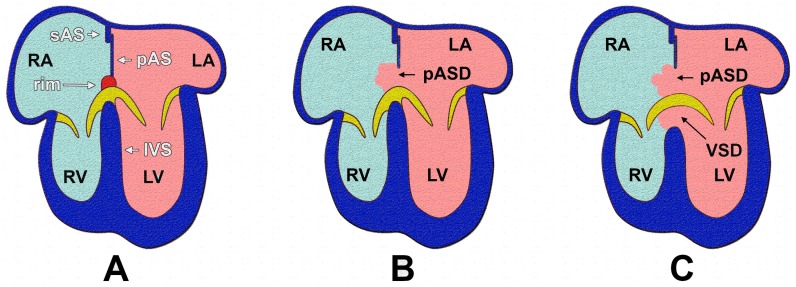
Anatomy of the atrioventricular septation complex in a human four-chambered heart with normal anatomy and in hearts with AVSDs. (**A**) shows a properly septated heart with a complete atrial and ventricular septum; (**B**) depicts a heart with an incomplete AVSD; and in (**C**) a heart with a complete AVSD is shown. Note that when a primary atrial septal defect is observed, the muscular rim at the base of the atrial septum is missing. IVS = interventricular septum, pAS = primary atrial septum, pASD = primary atrial septal defect, LA = left atrium, LV = left ventricle, RA = right atrium, RV = right ventricle, VSD = ventricular septal defect.

In the fully septated heart, the oxygenated blood in the left part of the heart is separated from the oxygen depleted blood in the right part of the heart by an atrial and a ventricular septum (Figure 1A). In the case of an AVSD, there is communication between the left and right part of the heart and there is the potential that oxygenated and de-oxygenated blood will mix. Based on the potential for shunting of blood between the left and right side of the heart, two major AVSD subtypes can be distinguished [18]. In “incomplete” AVSDs (Figure 1B), shunting is restricted to the atrial level via an ostium primum (or primary) atrial septal defect (pASD), while in “complete” AVSDs, in addition to a pASD, an inlet-type ventricular septal defect (VSD) is also found. In hearts with a complete AVSD (Figure 1C) left-right shunting of blood can therefore occur at atrial, as well as ventricular, levels (Figure 1).

### 1.3. Pathogenesis of AVSDs—AV Cushions vs. DMP

The pathogenesis of AVSDs has been the topic of research and discussion for many years. For a very long time, the accepted paradigm was that perturbation of the development or fusion of the endocardial AV cushions, the precursor tissues for the AV valve leaflets (see Section 2.1) was responsible for the creation of an AVSD. This common belief led to the introduction and frequent use of the term “endocardial cushion defect” to describe the abnormality [19,20]. At the time of writing this review, the majority of medical textbooks and popular medical websites still describe AVSDs as anatomic defects that arise from faulty development or inadequate fusion of the embryonic endocardial cushions. In recent years, a series of experimental studies in the mouse and a number of observations in the human heart have led to new insights into the etiology and pathogenesis of AVSDs. Specifically, these studies have linked abnormal development of the Dorsal Mesenchymal Protrusion (DMP) to the development of this congenital defect [21,22,23,24,25,26]. The most convincing experimental data linking dysmorphogenesis of the DMP with the pathogenesis of the pASD, the common component in all forms of AVSDs, (see Section 1.2), comes from studies with mouse models in which the development of the DMP has been perturbed using tissue-specific cre-recombinase mice in combination with mice carrying floxed alleles of genes playing a role in DMP development. In this contribution we will review the latest insights into the molecular and cellular mechanisms associated with the development of the DMP, revisit the role of the DMP in the formation of the AV mesenchymal complex, and discuss several conditions and models in which AVSDs are observed.

## 2. The Atrioventricular Mesenchymal Complex

To fully understand the role of the DMP in the pathogenesis of AVSDs it is important to describe all the components that participate in the formation of the atrioventricular mesenchymal complex and to discuss how these components relate to other tissues involved in atrioventricular septation.

### 2.1. The Mesenchymal AV Cushions

**Figure 2:**
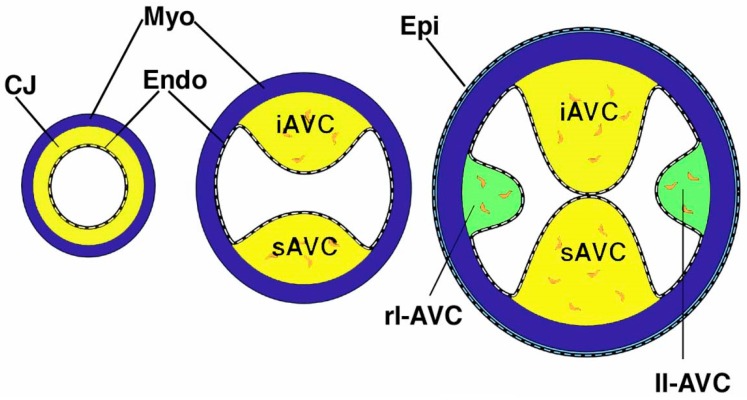
Development of the Atrioventricular Cushions. During formation of the heart tube, extracellular matrix-rich cardiac jelly (yellow) first accumulates between the endocardium and myocardium (**left**). During looping additional accumulation and endoEMT at the dorsal and ventral aspect of the AV canal results in formation of the major AV cushions (**middle**). The lateral cushions (green) form around the time the major cushions are fusing to separate left and right AV canal (**right**). CJ = cardiac jelly, Endo = endocardium, Epi = epicardium, Myo = myocardium, iAVC = inferior AV cushion, sAVC = superior AV cushion, ll-AVC = left lateral AV cushion, rl-AVC = right lateral AV cushion.

The development of the heart starts with the generation of the precardiac mesoderm forming two bilateral primary heart fields. These heart fields eventually fuse thereby creating the linear primary heart tube [27,28]. This heart tube consists of a myocardial outer mantle, an acellular matrix, often referred to as the cardiac jelly, and an inner endocardial tube [29]. The heart tube is initially suspended from the rest of the embryo over its entire length by the dorsal mesocardium. During cardiac looping this dorsal mesocardium largely disintegrates with the exception of the persisting dorsal mesocardium at the venous pole of the heart [30]. As the heart tube remodels, the atrial and ventricular chambers expand by a process sometimes referred to as ballooning [31]. During this process the chambers gradually lose most of the cardiac jelly between the myocardium and endocardium with the exception of the cardiac jelly at the atrioventricular junction (AVJ) and the outflow tract (OFT). In these parts of the heart, the cardiac jelly is accumulating in the subendocardial space resulting in the formation of prominent cushions. While in the early stage of their development these extracellular matrix-rich cushions do not contain any cells, a subsequent endocardial epithelial-to-mesenchymal transformation (endoEMT) generates a cohort of endocardially-derived mesenchymal cells that gradually migrates into and populates the cushions a process that is initiated around ED 9.5 in the mouse [32]. Within the AV junction, the two major (or midline) AV cushions form first. Around ED12.5, the major cushions fuse, thereby dividing the common AV canal into the left and right AV junction. In the left AV junction, forming the communication between the left atrium and left ventricle, the left AV valve (or mitral valve in the human) will develop, and in the right AV junction, connecting right atrium and right ventricle, the right AV valve (or tricuspid valve in the human) will form. The fused major AV cushions play a significant role in AV valve development as the aortic (or anterior) leaflet of the left AV valve, as well as the septal leaflet of the right AV valve derive from the fused major cushions [33,34]. Importantly, the fused major cushions also form the mesenchymal base on which the atrial septal complex develops [35]. After the formation of the major AV cushions a second set of cushions forms at the lateral AV junctions (Figure 2). These lateral AV cushions, which also become populated with mesenchymal cells as a result of endoEMT, are significantly smaller than the major cushions. The right lateral cushion eventually forms the parietal leaflet of the right AV valve, while the left lateral cushion forms the parietal (or mural/posterior) leaflet of the mitral valve.

### 2.2. The Mesenchymal Cap on the Primary Atrial Septum

The formation of the atrial septal complex involves the development of a myocardial primary and secondary atrial septum (pAS and sAS, respectively). Together with the mesenchymal tissues described in this section they are responsible for the formation of the atrial septal complex dividing the lumens of the left and right atria (see also Section 3). The process of atrial septation starts around ED10.5 with the formation of the pAS descending as a very thin sheet of atrial myocardium from the dorsal wall of the common atrium [36]. A specific feature of the pAS is that a mesenchymal cap is located along the entire leading edge. This cap develops as a result of endoEMT in a process similar to that seen in the AV and OFT cushions [33,35,36,37,38].

### 2.3. The Dorsal Mesenchymal Protrusion

**Figure 3:**
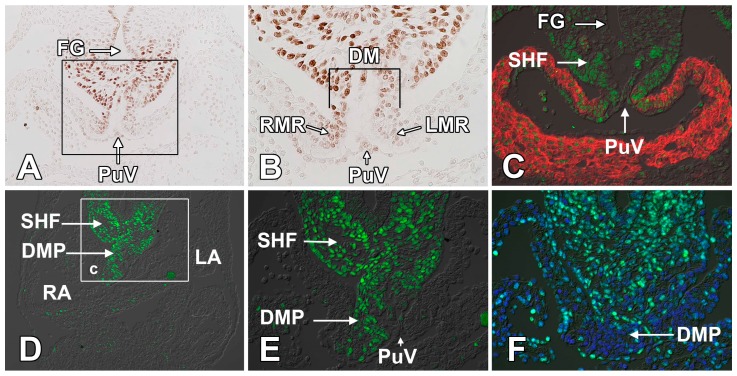
The Dorsal Mesocardium and the development of the Dorsal Mesenchymal Protrusion. (**A**,**B**) show a section of the venous pole of a mouse heart at 9.5ED stained for the expression of Isl1 ((**B**) is an enlargement of boxed area in (**A**)). Isl1 expression (brown nuclei) can be seen in the second heart field located between the dorsal mesocardium and the foregut. (**C**) is a section in the same area of a different heart at the same stage labeled for the expression of the myocardial marker Myosin Heavy Chain (red) and the expression of pSMAD1,5,8 (green) showing active BMP signaling. Note that the developing pulmonary vein is located in the midline of the dorsal mesocardium at this stage. The sections in (**D**,**E**) show the developing DMP at 10.5ED. (**D**,**E**) ((**E**) is an enlargement of the boxed area in (**D**)) show the penetrating DMP stained for Isl1 (green). (**F**) shows the DMP in a sister section stained for the proliferation marker ki67 (green) demonstrating that whereas there is active proliferation in the SHF itself, the level of proliferation in the SHF cells at the leading edge of the DMP is very low.

The third major component of the atrioventricular mesenchymal complex is the Dorsal Mesenchymal Protrusion (DMP) [36], sometimes also referred to as the “vestibular spine” or “spina vestibuli” [21,22]. The close relationship between the DMP and other developing atrial septal structures in the human heart was previously documented [36]. Unlike the mesenchyme found in the atrioventricular cushions and the mesenchyme in the cap on the pAS, the mesenchyme of the DMP is not endocardially-derived but instead derives from the Second Heart Field (SHF) [35,37]. A growing body of evidence shows that proper development of the DMP is critically dependent on the proliferation of the splanchnic mesoderm cells in the posterior SHF (pSHF) located between the dorsal mesocardium and the foregut [23,25,26] (Figure 3). In the normal situation, active proliferation of the pSHF results in expansion of this population between ED9.5 and ED10.5 ventrally into the common atrium using the dorsal mesocardium as its gateway. Failure of the DMP to develop is, at least partly, the result of compromised expansion of this cell population [24,25,33,35,39,40]. The formation of the DMP is not only critically important for the proper formation of the atrioventricular mesenchymal complex [35], the process also plays a very important role in determining the location of where the pulmonary vein(s) drain(s) into the heart. As the dorsal mesocardium becomes established at the venous pole of the heart, an endothelial invagination (also known as “pulmonary pit” [41]) can be seen in the dorsal wall of the common atrial cavity (Figure 3A–C). In human, this structure was first observed by Auer [42]. The endocardial lining of the pulmonary pit is contiguous with a non-lumenized endothelial strand, (also known as the “midpharyngeal endothelial strand” [43]) situated in the midline of the dorsal mesocardium. This endothelial strand represents the primitive pulmonary vein. As the DMP develops within the dorsal mesocardium it emerges to the right of the primitive pulmonary vein thereby translocating this structure toward the left of the midline (Figure 3). In subsequent stages, a myocardial differentiation of the mesenchyme flanking the developing pulmonary vein results in the incorporation of this structure into the myocardial wall of the left atrium [36].

## 3. Atrial Septation

**Figure 4:**
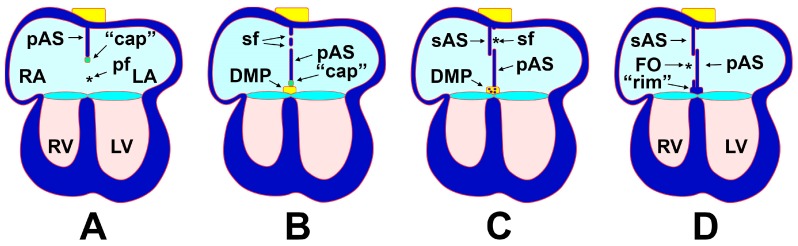
Atrial septation. This cartoon depicts the developmental events responsible for the formation of the atrial septal complex (see text). The yellow box in the back of the heart represents the posterior Second Heart Field giving rise to the DMP which in turn develops into the base of the atrial septal complexand becomes muscularized to form the muscular rim (**B**–**D**). The asterisk in (**A**) marks the primary foramen (also known as ostium primum); the asterisk in (**C**) marks the secondary interatrial foramen (also known as ostium secundum), which forms as a result of perforations in the upper part of the primary atrial septum (see (**B**)). DMP = dorsal mesenchymal protrusion, pf = primary foramen, FO = foramen ovale, LA = left atrium, LV = left ventricle, PAS = primary atrial septum, RA = right atrium, RV = right ventricle, “rim” = myocardial rim, sAS = secondary atrial septum.

The development of the atrial septal complex involves a complicated series of events. This includes the formation of the respective components of the atrioventricular mesenchymal complex (see above) and the development of a number of myocardial structures, in particular the myocardial component of the primary atrial septum (pAS) and the secondary atrial septum (sAS). To make matters more complex, it also involves the myocardial differentiation of the mesenchyme of the SHF-derived DMP during the formation of the muscular inferior rim. Given the complexity of all the events involved, it is virtually impossible to describe atrial septation as a simple series of consecutive events. As a result, the following description significantly simplifies the process (see also Figure 4).

### 3.1. The Formation of the Atrial Septal Complex—The Primary Atrial Septum

Atrial septation “starts” when the myocardial primary atrial septum (pAS; also referred to as the septum primum) starts to grow from the atrial roof into the common atrium. As the pAS develops it is in continuity with the left mesocardial reflection of the dorsal mesocardium [36]. It is therefore not entirely surprising that although the pAS more or less develops in the midline of the common atrium (Figure 4A), its molecular expression profile of the pAS identifies it as a structure with a molecular phenotype characteristic for left atrial structures. For instance, in the developing human heart, the pAS expresses relatively high levels of the B-isoform of Creatine Kinase (CK-B) [36], a feature characteristic of the developing left atrium, whereas studies in the mouse have shown that the pAS expresses Pitx2c, a transcription factor found in the left, but not the right, atrium [44]. In the early stages of atrial septation the left and right atrium continue to be in open communication with each other (Figure 4A). The space under the pAS is called the primary foramen (or ostium primum). As mentioned, on the leading edge of the pAS is located an endocardially-derived mesenchymal cap.

While the mesenchyme found in the cap and the mesenchyme of the DMP has different origins (i.e., endocardially-derived vs. SHF derived), the two mesenchymal tissues are contiguous. The development of the pAS and that of the DMP are therefore closely related [35]. During normal development, the mesenchymal cap, the DMP, and the major AV cushions will eventually fuse and close the primary foramen. The body of mesenchyme formed after the fusion of the mesenchymal cap, DMP, and AV cushions is known as the AV mesenchymal complex [35]. Eventually, the mesenchyme of the DMP, but not the mesenchyme of the cap and cushions, undergoes a myocardial differentiation (Figure 4C), a process during which the transcription factor Isl1 (characteristically expressed in SHF cells) is downregulated and the transcription factor Nkx2.5 (expressed in virtually all myocardial cells) becomes expressed in these SHF derived cells. In the formed heart, the muscularized DMP can be recognized as the inferior muscular rim at the base of the atrial septum[36,45]. Before the descending pAS and AV mesenchymal complex close off the primary foramen, a secondary foramen (or foramen secundum) is developing within the pAS itself (Figure 4B).

### 3.2. The Formation of the Atrial Septal Complex–The Secondary Atrial Septum

The secondary atrial septum (sAS) develops later than the pAS (Figure 4C) and is found at the boundary between myocardium with a left atrial phenotype and myocardium with right atrial characteristics in the atrial roof to the right of the pAS. Throughout fetal life the communication between the left and right atrium remains functionally open to allow the right to left shunt essential in the fetal circulation. After birth, the pAS typically fuses with the sAS (Figure 4D), thereby physically separating both atrial chambers [36,46]. In approximately 25 percent of the general population this fusion does not take place resulting in a patent foramen ovale (PFO), which, in general, does not cause any problems. However, it is important to note that the incidence of PFO is 40 to 50 percent in patients who suffer from a stroke of unknown cause, a condition typically referred to as a cryptogenic stroke where the PFO allows the passage of small blood clots that can lodge themselves in and block arteries of the brain.

## 4. Molecular Regulation of DMP Development

Insights into molecular mechanisms that govern the cellular events in the (posterior) SHF that allow proper DMP development and its maturation are slowly emerging. In Section 4.1 through Section 4.7 we will provide an overview of recently published studies in the mouse that have improved our understanding of how the development and growth of the pSHF and DMP are regulated and how perturbation of DMP development plays a role in the pathogenesis of AVSDs. In Section 5 we have schematically summarized some of these insights.

### 4.1. The BMP Signaling Pathway and AVSDs

**Figure 5:**
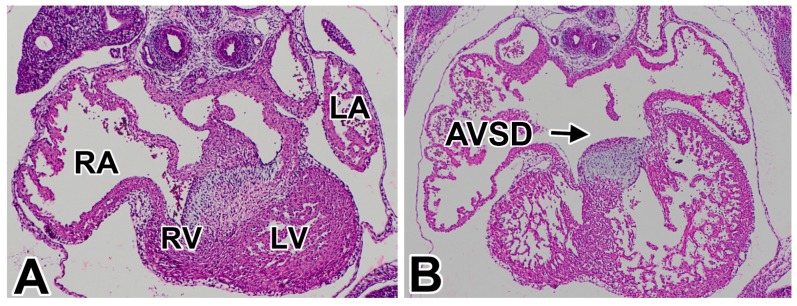
Atrioventricular septal defect in Mef2C-AHF-Cre;Alk3^fl/fl^ mouse at ED13.5 Histological analysis of control (**A**) and SHF-specific Alk3 knockout (**B**) mouse embryos at ED13.5 shows that while the atrioventricular valvuloseptal complex is complete in the control specimen (**A**) the deletion of Alk3 from the SHF has resulted in an incomplete AVSD in the SHF-specific Alk3 knockout embryo (**B**). AVSD = atrioventricular septal defect, LA = left atrium, LV = left ventricle, RA = right atrium, RV = right ventricle.

Bone Morphogenetic Proteins (BMPs) play an important role in cardiovascular development. At least 6 BMP isoforms are expressed in the heart, including BMP2, BMP4, BMP5, BMP6, BMP7, and BMP10 [47,48,49,50]. Of these BMP isoforms, BMP2 is the most frequently studied specifically because of its importance in AV cushion development. At the critical stages of AV cushions formation, BMP2 is expressed at high levels in the AV myocardium [48] where it plays an essential role in the regulation of endoEMT [51]. Mice that do not express BMP2 die before E12.5 with severe defects in AV cushion development [51,52]. BMP4 is also critically important for normal heart development [53,54,55]. Mice deficient for this BMP isoform show early embryonic lethality [56] while BMP4 hypomorphic mice, i.e., mice expressing severely reduced amounts of BMP4, die within one week after being born. The importance of BMP4 in AV septation was first demonstrated in a study by Jiao et al., who described that in the hypomorphic BMP4 mouse 100% of the offspring was characterized by having an AVSD [57]. The same study also reported that myocardial-specific deletion of BMP4 results in AVSDs as well [57]. The observations in the paper from Jiao and colleagues, in combination with our own BMP expression studies showing that BMP4 is abundantly expressed in the myocardial walls of the dorsal mesocardium (also known as the mesocardial reflections) but not in the myocardial AV junction, led us to investigate the role of BMP signaling in the development of the DMP in more detail [23]. The BMP4-expressing mesocardial reflections flank the space through which the expanding pSHF protrudes as the DMP into the common atrial cavity [18,33]. The working hypothesis for the study was that the BMP4 expression in the mesocardial reflections was critically important for the development of the DMP. In the paper we demonstrated that the pSHF population that eventually will form the DMP expresses BmpR1A/Alk3, a BMP receptor known to interact with BMP4. The pSHF cells also stained positive for pSmad1,5,8 indicating active BMP signaling (see also Figure 2C). We then proceeded with generating a SHF-specific Alk3 knockout mouse using the Mef2C-AHF-Cre mouse line [58] in combination with a floxed Alk3 mouse. The SHF-specific deletion of Alk3 resulted in impaired formation of the DMP and a completely penetrant incomplete AVSD (ostium primum defect) phenotype at stages ED13.5 to 15.5 (Figure 5). Analysis of Mef2C-AHF-Cre;Alk3^fl/fl^ mutants at E10-10.5 revealed decreased proliferative index of SHF cells and, consequently, a reduced number of pSHF cells at the cardiac venous pole [23]. Thus, based on these observation it can be concluded that BMP signaling is required for, or is involved in the regulation of, the expansion of the SHF-derived DMP progenitor population at the cardiac venous pole, a process essential for the proper development of the DMP and the formation of atrioventricular complex.

### 4.2. The Wnt/β-Catenin Signaling Pathway and AVSDs

The Wnt/β-catenin signaling pathway plays a crucial role in many developmental events. During cardiogenesis Wnt/β-catenin signaling is involved in the regulation of the formation of the SHF-derived outflow tract, right ventricle, and the venous pole [59,60]. Deletion of β-catenin with the Isl1-Cre mouse (which like the Mef2c-AHF-Cre mouse is driving Cre-expression in the SHF) results in significant down regulation of a number of β-catenin downstream genes including Isl1, Tbx3, Wnt11, and Shh. It also results in the reduction of proliferation of SHF-derived cardiac progenitor cells and embryonic lethality. Cardiovascular abnormalities observed in the Isl1-Cre; β-catenin mouse include pharyngeal arch defects and AVSDs [61]. A few years ago, an outstanding study by Tian et al. [25] addressed the role of Wnt2 in the development of the venous pole. In their paper the authors demonstrated that normal expression of Wnt2 is essential for proper development of the posterior SHF and the DMP [25]. Data presented in this study show that Wnt2 and the Wnt target genes Lef-1 and axin2 are expressed in the SHF/DMP-precursor cells at the critical stage of DMP development (ED9.5–ED10.5) [25,62]. Significance of Wnt2 expression for AV septation was shown in studying the Wnt2 knockout mouse. Approximately 85% of Wnt2 knockout offspring generated in this study died at birth and histological analysis of Wnt2 knockout specimens revealed the presence of AVSDs [25]. Close inspection of the SHF/DMP-precursor population at the venous pole of Wnt2 knockout embryos showed reduced expression of Lef1 and axin2 as well as a reduction in the level of Fgf10, a direct target of Wnt/β-catenin signaling. Furthermore, the proliferative activity in the SHF/DMP-precursor population of the Wnt2 knock-out mouse at E9.5 was significantly lower than the one observed in control embryos [25,60]. The proliferation defect and associated failure of the DMP to properly develop resembles what is observed in the Mef2C-AHF-Cre;Alk3^fl/fl^ [23] (see also above). To determine the requirement of Wnt2 activity in development and cardiac morphogenesis the authors used LiCl, a pharmacological inhibitor of Gsk-3b and a strong activator of Wnt/β-catenin signaling. Administration of LiCl to pregnant female mice carrying Wnt2 knockout embryos at ED8-10, led to a drastic increase in the survival rate of Wnt2 knockout offspring, an elevation of Lef1 expression in the posterior SHF, restoration of SHF cell proliferation, and a rescue of the AVSD phenotype [25].

### 4.3. The (Sonic) Hedgehog Signaling Pathway and AVSDs

**Figure 6:**
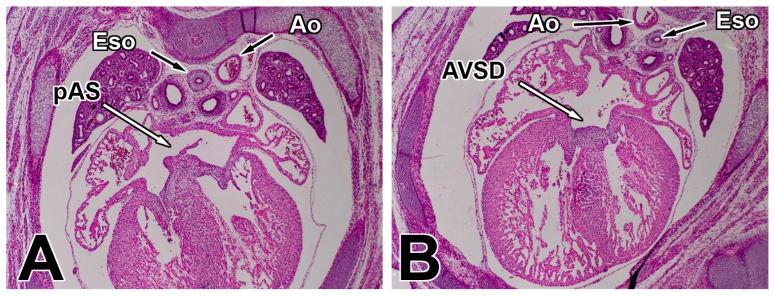
Atrioventricular septal defect in Mef2C-AHF-Cre;Smo^fl/fl^ mouse at ED15.5. This figure shows a control (**A**) and SHF-specific Smo knockout (**B**) mouse embryo at ED15.5. The image in (**A**) demonstrates normal anatomy of the atrioventricular septal complex, whereas the conditional knockout embryo in (**B**) is characterized by an incomplete AVSD. Note also that the dorsal aorta in (**A**) is located in its normal position to the left of the esophagus and the embryonic midline while in (**B**) the dorsal aorta is located to the right of the esophagus. Ao = dorsal aorta, AVSD = atrioventricular septal defect, Eso = esophagus, pAS = primary atrial septum.

Hedgehog (Hh) proteins are secreted morphogens that play a very important role in patterning events in the developing embryo. The family of Hh proteins includes Sonic hedgehog (Shh), Desert hedgehog (Dhh) and Indian hedgehog (Ihh). Hh signaling involves two transmembrane bound proteins that in vertebrates are associated with primary cilia [63]. Binding of an Hh ligand to Patched (Ptch1) abolishes an inhibitory effect on Smoothened (Smo) allowing signal transduction via Gli transcription factors [64]. In the context of cardiac development, Shh is the most frequently studied Hh variant. Several independent studies have shown that Shh signaling is involved in controlling the contribution of the SHF to the developing outflow tract, the cardiac venous pole, and the atrioventricular septal complex [24,39,65]. It is important to note that even though Shh knockout mice display severe cardiac defects, Shh itself does not seem to be expressed within the heart itself [66,67], indicating that tissues outside the heart proper must be involved in Shh-dependent regulation of heart development. One of the extra cardiac tissues secreting Shh is the ventral pharyngeal endoderm [65]. The Shh expressing ventral pharyngeal endoderm is juxtaposed to the SHF/DMP-precursor cell population located between the foregut and the heart and in close proximity to the cardiac arterial and venous poles. In a very elegant study, Goddeeris and colleagues showed that conditional deletion of Shh with a Nkx2-5-Cre mouse, directing expression of cre-recombinase to a variety of tissues including the pharyngeal endoderm, resulted in single outflow tract (i.e., persistent truncus arteriosus; PTA), as well as a fully penetrant AVSD phenotype [65], defects that were also reported by Washington Smoak and co-workers to be present in the Shh knockout mouse [66]. In another study on the role of Hh signaling in the developing heart, Goddeeris and colleagues conditionally deleted Smo from the SHF/DMP-precursor cell in heterozygous Smo knockout mice using the Mef2c-AHF-Cre mouse model, thereby generating Mef2c-AHF-Cre;Smo^fl/−^ offspring in which Shh signaling was abolished in the SHF/DMP-precursor cells. This approach led to perturbation of DMP formation and resulted also in a fully penetrant (partial) AVSD phenotype [24]. In a recent paper, we generated a SHF-specific conditional knockout for Smo (Mef2c-AHF-Cre;Smo^fl/fl^) and found partial AVSDs in approximately 2/3 of all specimens inspected (Figure 6) [26]. Proliferation studies in this model showed that interfering with the Hh signaling pathway significantly decreased the level of proliferation in the SHF/DMP-precursor cell population which, in turn, resulted in reduced numbers of Isl1-expressing SHF cells and failure of the DMP to develop properly. Importantly, we also determined that, by, assessing the expression level of Lef1 and Axin2, two downstream targets of the Wnt/β-catenin pathway, this pathway was compromised in the SHF/DMP-precursor cells of the Mef2c-AHF-Cre;Smo^fl/fl^ embryos. Thus, interaction between Shh (expressed in, and secreted from pharyngeal endoderm) and the Shh receptor Smo (expressed in the SHF/DMP-precursor cell population) is essential for development of the DMP and, hence, for proper AV septation. Importantly, we found that the expression of two downstream targets of the Wnt/β-catenin pathway, Lef1 and Axin2, was also reduced in the SHF/DMP-precursor cells of Mef2c-AHF-Cre;Smo^fl/fl^ embryos and concluded that this Wnt/β-catenin pathway was compromised in the SHF/DMP Mef2c-AHF-Cre;Smo^fl/fl^. To determine whether the reduced Wnt/β-catenin signaling observed in the Mef2c-AHF-Cre;Smo^fl/fl^ embryos could be responsible for the AVSDs found in these specimens we adapted the protocol used by Tian et al. [25] (see also Section 4.2) and administered, LiCl, the pharmacological activator of the Wnt/β-catenin pathway, to fertilized female mice carrying Mef2c-AHF-Cre;Smo^fl/fl^ embryos. This resulted in restoration of proliferation of the SHF/DMP-precursor cell population and partial rescue of the AVSD phenotype. Based on these observations we concluded that the Wnt/β-catenin pathway is either acting downstream of, or parallel to, the Shh pathway in the regulation of atrioventricular septation.

### 4.4. Pdgf and Pdgf Receptors and AVSDs

Platelet-derived growth factors (Pdgfs) and their receptors (Pdgfrs) are important for cardiac development. For instance, Pdgf-B ligand knockout mice have multiple cardiac defects, including VSDs, double outlet right ventricle (DORV), aortic arch abnormalities, and sporadic AVSD [68]. Pdgfrα is expressed in Neural Crest Cells (NCCs) including the cardiac NCCs that contribute to outflow tract development [69,70] and also in the SHF/DMP-precursor cell population and the DMP [71,72,73]. An integrated approach using human genetics and mouse models showed association between dysregulation of Pdgfrα with Total Anomalous Pulmonary Venous Return (TAPVR), a serious congenital heart defect [73]. Additional studies with Pdgfrα knockout mouse models have demonstrated the presence of a spectrum of other abnormalities including common arterial trunk (or persistent truncus arteriosus), DORV, and AVSD [74]. As in other models described above, Bax et al. [74] found that the AVSD in the Pdgfrα knockout mouse was associated with hypoplasia of the DMP. Interestingly, they also reported elevated expression levels of the transcription factor Nkx2.5, a transcription factor typically expressed in embryonic and differentiated cardiomyocytes. In an earlier paper from our lab we have shown that Nkx2.5 is switched on in cells of the DMP as they undergo mesenchymal-to-myocardial differentiation around E14 [40]. If the gradual increase in Nkx2.5 expression in the DMP is the driving force behind the muscular differentiation of the DMP mesenchyme after the formation of the AV mesenchymal complex, and if Nkx2.5 expression is indeed elevated in the pSHF of the Pdgfrα knockout mouse, then premature and ectopic myocardial differentiation of the pSHF cell population should be considered as a possible mechanism involved in inhibiting normal DMP formation and causing AVSD. In this context it is interesting to note that premature myocardial differentiation of pSHF has also been suggested to be involved in the pathogenesis of AVSDs in Mef2c-AHF-cre;Smo^fl/−^ embryos [24], albeit that premature myocardial differentiation was not observed in our analysis of Mef2c-AHF-Cre;Smo^fl/fl^ embryos [26].

### 4.5. Tbx5 and AVSD

Holt-Oram Syndrome (HOS) is a serious condition caused by haploinsufficiency of the transcription factor Tbx5 [75,76]. Patients suffering from HOS present with a spectrum of congenital malformations, including limb and cardiac defects. The Tbx5 knockout mouse is an animal model for HOS. In this mouse model ASDs and AVSDs are found in approximately 40% of the haploinsufficient offspring [77]. Within the pSHF, Tbx5 is co-expressed with Gli1, a member of the Gli transcription factor family and responsible for Shh signal transduction [77]. Conditionally deleting Tbx5 from the SHF with two SHF Cre-mouse models (Mef2c-AHF-Cre and Gli1-Cre-ERT2) revealed that Tbx5 expression in the pSHF is critical to the development of the DMP as in both conditional Tbx5 knockout models a fully penetrant incomplete AVSD phenotype (ostium primum defect) was found [77]. To determine how atrial septation is perturbed in these models, BrdU incorporation assays were performed at ED9.5. This revealed a significant decrease in proliferation of the SHF/DMP-precursor cell population, similar to what is documented in other models with AVSDs. Similar to what is seen in these other models, TUNEL staining did not indicate an increase in apoptosis indicating that the SHF/DMP-precursor cells require Tbx5 for proliferation but not survival. Quantitiative analysis of markers for Shh signaling in the Mef2c-AHF-Cre;Tbx5 embryos revealed a significant reduction in the expression of the Shh receptor Ptch1 (see Section 4.2) and Gli1, suggesting that Shh signaling is downstream of Tbx5 in the regulation of pSHF and DMP development. To test this hypothesis, Xie and colleagues created hetero- and homozygote SHF-specific Tbx5 mutants that, in addition, also carried a constitutively activated Smo mutant allele (SmoM2) under the control of the SHF-specific Cre construct. This approach allowed SHF-specific activation of Hh-signaling in the SHF cells in which the expression of Tbx5 was abolished. The SHF-specific Hh-signaling led to a complete rescue of atrial septation in conditional SHF Tbx5 heterozygotes and a five-fold reduction in homozygous SHF Tbx5 mutants suggesting that Tbx5 is acting upstream or in parallel to Hh-signaling in SHF-dependent atrial/atrioventricular septation.

### 4.6. Primary Cilia and AVSDs

Primary cilia are solitary organelles protruding from the cell surface of most mammalian cell types. The core structure of primary cilia is the ciliary axoneme, which consists of nine doublet microtubules that extends, via a transition zone, from a microtubule-based basal body. Intraflagellar transport (IFT) isoforms are proteins that play crucial roles in the formation, maintenance, and function of cilia and allow the trafficking of particles up and down along the axoneme. Two IFT complexes can be distinguished; the IFT-A complex and the IFT-B complex. The proteins in the IFT-A complex are involved in controlling retrograde ciliary trafficking, whereas the proteins found in the IFT-B complex are required for anterograde trafficking from the base to the tip of the cilium. Ciliary “dysfunction” has been associated with a spectrum of human congenital malformations, including defects found in the cardiovasculary system, such as AVSDs. A comprehensive study of a mouse model carrying a hypomorphic mutation of the IFT protein Ift172 (Ift172^avc1^/Ift172^avc1^) showed AVSDs in 100% of the mutants analyzed [78]. A similar observation was reported for the cobblestone (cbs) mutant, a mouse carrying a hypomorphic allele for Ift88 (also known as Polaris) as cbs/cbs mutants are also characterized by having a complete AVSD [79]. Interestingly, a few years ago, Ripoll and colleagues published a paper demonstrating an association between AVSDs and other congenital heart malformations found in Down syndrome patients with an altered ciliome [80]. Cilia are important in a number of signal transduction and molecular pathways including the Wnt/β-catenin signaling pathway, the PDGF pathway, the Notch pathway, and the Hedgehog signaling pathway. The Hh pathway is by far the one most frequently studied. Hh signaling is initiated when a secreted Hh ligand (typically Shh) binds to the membrane receptor Patched (Ptc1) localized on the distal end of the axoneme. This interaction alleviates inhibition of the transmembrane protein Smoothened (Smo; see Section 4.2) by Ptc1. Smo then activates the transcriptional activator Gli2, thereby transmitting the Shh signal to the nucleus. The Shh signaling pathway is critically dependent on intraflagellar transport which acts downstream of Ptc1 and Smo but upstream of the Gli transcriptional activity. Thus, primary cilia, present on the cells of the posterior SHF, may play a pivotal role in atrioventricular septation through the regulation of the Shh pathway and associated molecular and cellular mechanisms. A recent study from the Lo laboratory found 34 cilia-related genes in a recessive forward genetic screen in fetal mice mutagenized with ethylnitrosourea (ENU) to identify candidate genes that might be play important roles in the pathogenesis of CHD. One of the genes identified was Cc2d2a, which was found to cause AVSD [81]. In human, mutations in CC2D2A are associated with Joubert syndrome, a ciliopathy associated with cerebellar abnormalities and other birth defects. Combined, the studies listed here show that primary cilia, and cilia-related pathways, are very important in atrioventricular septation.

### 4.7. AVSDs and Heterotaxy Syndrome

While in the normal anatomical situation in humans all internal organs, including the liver, lungs, kidneys and the heart, have distinctive left/right characteristics (a condition known as situs solitus), in heterotaxy this left/right laterality of the internal organs is disturbed (a condition referred to as situs ambiguous). Heterotaxy is a severe condition and has a reported incidence of one in ten thousand and at least 3% of individuals with congenital heart defects are patients suffering from heterotaxy [82]. The two extremes encountered in heterotaxy can be described and defined by the morphology of the atria [83]. Left Atrial Isomerism (LAI) is the situation when both the left as well as the right atrium have pronounced left atrial characteristics. The condition in which both atria have features normally seen in the right atrium is known as Right Atrial Isomerism (RAI). Importantly, approximately two-third of all patients with LAI or RAI have an AVSD. Long-term outcome for patients with isomerism has historically been very poor [84]. Heterotaxy can be caused by defects in the motile cilia that are present in the (primitive) node, a structure found in the early stages of embryonic development. Motile cilia are required for left/right body axis determination. At ED8.0 in mouse, the nodal cilia facilitate a leftward flow of extraembryonic fluid which, in turn, leads to the asymmetrical distribution of molecular cues ultimately resulting in the establishment of the left/right asymmetry including normal asymmetrical cardiac morphogenesis [85]. Thus, gene mutations that negatively affect the function of the motile cilia at early embryonic stages may cause AVSDs in a different way than mutations that affect the development of the SHF in slightly more advances stages a concept that was recently very nicely presented in a paper from the Moskowitz lab [86], in which the authors describe how a mutation in Dnah11, a cilia motility gene, leads to heterotaxy (RAI, as well as LAI) with associated AVSDs, whereas a mutation in Mks1, a centrosomal protein essential for primary cilia formation, leads to 100% AVSDs in hearts with normal left/right patterning.

## 5. Etiology and Pathogenesis of AVSDs—Conclusions and Future Directions

**Figure 7:**
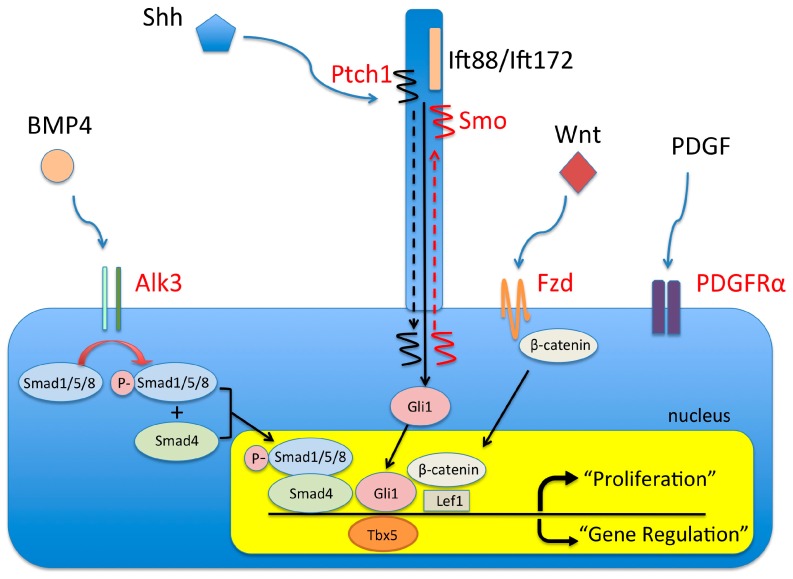
Model of a posterior SHF-DMP precursor cell. This simplified cartoon of a DMP precursor cell with a protruding primary cilium shows pathways and factors known to be involved in pSHF/DMP-dependent atrioventricular septation.

Studies described in this review show that failure of proper development of the SHF-derived DMP leads to a primary atrial septal defect (pASD), the common septal defect seen in complete and incomplete AVSDs. This new insight has led to a paradigm shift in the understanding of AVSDs as the prevailing paradigm for many years was that failure of proper development of the AV cushions was the sole defect responsible for AVSDs [20]. Furthermore, we have discussed in this contribution a series of studies focusing on the roles of a number of well-known pathways and transcription factors in the context of the development of the DMP and the etiology of AVSDs. In Figure 7 we present a simplified model of a SHF/DMP-precursor cell with the associated pathways and factors. While this new insight into the role of the SHF and the DMP has changed our understanding of the events leading to AVSD, it does not, however, provide answers to all the questions that exist regarding the pathogenesis of AVSDs. One of the questions that remains to be resolved relates to the difference in etiology of complete vs. incomplete AVSDs. Based on what we currently know about the pathogenesis of incomplete AVSDs in the mouse models studied, the current working hypothesis is that isolated pASD (incomplete AVSD) result from (genetic) defects that only affect the development of the SHF/DMP-precursor cell population. We believe that the pathogenesis of complete AVSD might require developmental perturbations in the SHF/DMP-precursor cell population, as well as abnormal development of cardiac tissues with a different developmental origin, such as the AV cushions and/or the ventricular myocardium.
